# An overview of human single-cell RNA sequencing studies in neurobiological disease

**DOI:** 10.1016/j.nbd.2023.106201

**Published:** 2023-06-13

**Authors:** T. Jordan Walter, Robert K. Suter, Nagi G. Ayad

**Affiliations:** Georgetown University, Lombardi Comprehensive Cancer Center, 3970 Reservoir Rd NW, Washington D.C. 20007, USA

**Keywords:** Single-cell RNA sequencing, Neurobiological, Human, Brain, Postmortem, Organoids

## Abstract

Neurobiological disorders are highly prevalent medical conditions that contribute to significant morbidity and mortality. Single-cell RNA sequencing (scRNA-seq) is a technique that measures gene expression in individual cells. In this review, we survey scRNA-seq studies of tissues from patients suffering from neurobiological disease. This includes postmortem human brains and organoids derived from peripheral cells. We highlight a range of conditions, including epilepsy, cognitive disorders, substance use disorders, and mood disorders. These findings provide new insights into neurobiological disease in multiple ways, including discovering novel cell types or subtypes involved in disease, proposing new pathophysiological mechanisms, uncovering novel drug targets, or identifying potential biomarkers. We discuss the quality of these findings and suggest potential future directions and areas open for additional research, including studies of non-cortical brain regions and additional conditions such as anxiety disorders, mood disorders, and sleeping disorders. We argue that additional scRNA-seq of tissues from patients suffering from neurobiological disease could advance our understanding and treatment of these conditions.

## 1. Introduction

Neurobiological disorders are highly prevalent medical conditions that contribute to significant morbidity and mortality (Group, 2017; Kessler et al., 2005). They are often treated with pharmacological agents, although resistance and adverse side-effects necessitate novel treatment strategies (Hilt et al., 2014; Howes et al., 2022; Kennedy et al., 2014; Laxer et al., 2014). Advancing our understanding of the underlying neurobiology of these disorders is important for therapeutic development. One new technique that is improving our understanding of these conditions is single-cell RNA sequencing (scRNA-seq). scRNA-seq has yielded unprecedented insight into the transcriptomic profiles of both healthy and diseased tissue. Whereas methods such as bulk RNA sequencing assay average gene expression across millions of cells, scRNA-seq measures gene expression in individual cells, thereby yielding a richer understanding of tissue function. scRNA-seq may aid in discovering novel cells involved in disease, proposing new pathophysiological mechanisms, uncovering novel drug targets, or identifying potential biomarkers. In addition to measuring cell-specific expression of individual genes, scRNA-seq can also identify gene regulatory networks (sets of co-regulated genes), potentially providing further insight into pathophysiology (Fiers et al., 2018). Such technologies could also advance personalized medicine, as we may be able to identify distinct patterns of single-cell gene expression in different individuals. Overall, scRNA-seq has the potential to enhance our understanding and treatment of neurobiological disease.

sc-RNA-seq studies in neurobiological disease take one of two main approaches: sequencing postmortem brain tissue or brain organoids generated from peripheral human cells. The brain organoids in these studies are usually generated from induced pluripotent stem cells derived from peripheral blood cells from patients with neurobiological conditions. In this review, we sought to survey scRNA-seq of tissues from patients suffering from neurobiological conditions, including central nervous system neurological disorders and psychiatric disorders. In addition to scRNA-seq, we also reviewed single-nucleus RNA sequencing (snRNA-seq), which uses isolated nuclei rather than whole cells to profile gene expression. While scRNA-seq measures transcripts in both the cytoplasm and the nucleus, snRNA-seq measures transcripts only in the nucleus. Frozen tissues are more amendable to snRNA-seq, which is why postmortem human brain studies generally use snRNA-seq as opposed to scRNA-seq. We identify reports covering a range of conditions, including epilepsy, cognitive disorders, substance use disorders, and mood disorders. In addition to summarizing these findings and identifying common themes, we also discuss the quality of these studies and identify areas ideal for further research and potential future directions. Further, we highlight ways in which prior sc- or snRNA-seq studies have provided novel insights into neurobiological disease. Finally, we argue that additional sc- or snRNA-seq research of neurobiological disease could advance our treatment of these conditions.

## 2. Studies in Post-Mortem brain tissue

### 2.1. Epilepsy

A few studies have performed scRNA-seq on brain samples from epilepsy patients. One study examined single nucleus gene expression specifically in neuronal cells of the temporal cortex from temporal lobe epilepsy patients (Pfisterer et al., 2020). The authors measured gene expression in several subtypes of principal neurons and GABAergic interneurons. They noted transcriptomic differences in nearly all subtypes of neurons when comparing epilepsy versus control samples. For example, genes encoding subunits of the excitatory AMPA receptor (GRIA1) were upregulated in several subtypes of principal neurons. Furthermore, genes encoding enzymes in GABA synthesis (e.g. – GAD1, GAD2) were decreased in some interneuron subtypes. The authors also performed gene network analyses, finding that networks involved in hyperexcitability, such as AMPA receptor subunits, glutamate receptor subunits, and voltage-gated sodium channels were dysregulated in epileptic tissue. Another study examined immune cells isolated from brain samples of pediatric epilepsy patients (Kumar et al., 2022). They found increased pro-inflammatory gene expression (e.g. - IL1B, IL1A, TNF, CCL4, and CCL2) in microglia from epilepsy samples compared to controls from people without a neurological disorder. The authors also found transcripts indicative of immune cell infiltration in epilepsy, including CD4^+^ T cells, CD8^+^ T cells, B cells, and NK cells. Many of the immune cells showed an inflammatory and cytotoxic phenotype, as well as expression of genes such as adhesion molecules and chemokine receptors that facilitate infiltration. Overall, these studies found dysregulated expression of hyperexcitability genes in neurons and increased expression of inflammatory genes in immune cells from tissue of epilepsy patients.

### 2.2. Multiple sclerosis

One study performed snRNA-seq of the white matter of postmortem samples from multiple sclerosis (MS) patients (Jäkel et al., 2019). They focused their study on oligodendrocytes and found seven different transcriptionally defined oligodendrocyte subtypes. The authors observed fewer oligodendrocyte progenitor cells (OPCs) in MS samples, as well as reduction of some oligodendrocyte subtypes and enrichment of others in MS samples. There was also upregulation of myelin-associated genes, such as MBP, CNP, and MAG, in MS oligodendrocytes compared to control oligodendrocytes. The authors also observed that cells from different types of MS lesions (e.g. – active lesions, chronic inactive lesions, remyelinated lesions, etc.) were transcriptionally distinct, illustrating that MS lesion subtypes may be identifiable by different marker genes. For example, WWOX was reduced in chronic active lesions and NKAIN2 was lower in remyelinated regions. These data demonstrate differential gene expression in MS brain tissue compared to normal brain tissue, as well as differential gene expression between subtypes of MS lesions.

### 2.3. Alzheimer’s

Several studies have performed scRNA-seq or snRNA-seq on the postmortem brains of Alzheimer’s disease (AD) patients. For a more comprehensive review of these studies, see (Luquez et al., 2022; Murdock and Tsai, 2023). Here we focus on sequencing of transcriptomes of all cell types in brain parenchyma. One study examined the entorhinal cortex of brains from people with AD and non-diseased age-matched controls (Grubman et al., 2019). They noted upregulation of stress response genes (e.g. – mitochondrial genes, heat shock genes, and molecular chaperone genes) across multiple cell types. In AD samples, neurons showed downregulation of GABA receptor (GABRA2, GABRB1), glutamate receptor (GRIA2, GRID2), and neurexin (NRXN1, NRXN3) genes, consistent with the loss of synapses seen in AD. Genes related to glial cell development and differentiation (such as BIN1 and CNTN2) were upregulated in astrocytes and oligodendrocytes, possibly as a compensatory response to myelin loss. Endothelial cells upregulated genes involved in cytokine secretion and immune responses (HLA-E, MEF2C, and NFKBIA) in AD. The authors also used their data to build gene regulatory networks and investigate the relationships between transcription factors and downstream target genes associated with AD. For example, they found upregulation of the TFEB gene in AD astrocytes compared to control astrocytes. TFEB encodes a transcription factor that is a master regulator of lysosomal function and acts upstream of several genes identified by genome-wide associated studies (GWAS) to be associated with AD. These genes, including POLN, STK32B, EDIL3, AKAP12, HECW1, WDR5, LEMD2, BIN1, and CLDN11, were also upregulated in AD astrocytes.

Mathys et al. also performed snRNA-seq of post-mortem brains of AD patients and controls with no pathology (Mathys et al., 2019). Their study was different from that of Grubman et al. in that they examined the prefrontal cortex rather than the entorhinal cortex. They found that excitatory neurons and oligodendrocytes in AD showed upregulated myelination-related genes (e.g. - LINGO1). Microglia and astrocytes showed upregulation of genes such as CD81 and GFAP, respectively, suggesting activation of these cell types in AD. The authors also compared cell-specific changes in gene expression in early- versus late-stage AD samples. Like Grubman et al., they found upregulation of protein folding genes, molecular chaperone genes, and global stress response genes in multiple cell types in late-stage AD samples vs early-stage AD samples. Importantly, it is worth noting that a recent preprint reanalyzed this data set using best-practice approaches and found different results (Murphy et al., 2023), underscoring the importance of re-assessing datasets based on consistently-evolving platforms.

Another study examined how different variants of the TREM2 gene affected single-nucleus gene expression in the postmortem brains of people with AD (Zhou et al., 2020). TREM2 is a microglial receptor. Mutations in this receptor affect the ability of microglia to surround the plaques associated with AD, leading to greater neuronal dysfunction. The R62H variant and R47H variant of TREM2 increase the risk of AD compared to the common variant of TREM2. The results showed differential gene expression in TREM2-R62H microglia and TREM2-R47H microglia from AD samples compared to TREM2-common variant microglia from AD samples. The authors noted increased expression of genes involved in oxidative stress and lipid metabolism and decreased expression of genes involved in growth-factor signaling and neural connectivity in AD with TREM2-R47H samples. These results demonstrate that microglial transcriptomes differ in AD depending on the variant of TREM2 expressed. Another study by Leng et al. performed snRNA-seq of the postmortem brains of AD patients to characterize changes in the relative abundance of cells as AD progressed (Leng et al., 2021). They used samples from Braak stages 0, 2, and 6, which represent pre-, early, and late stages of tau neurofibrillary pathology, respectively. The authors observed reduced excitatory neurons in the entorhinal cortex and superior frontal gyrus in Braak stage 6 AD compared to controls. Alternatively, there were no changes in inhibitory neuron transcripts in either brain region as AD progressed. There was a trend toward increased microglial counts in the entorhinal cortex with AD progression, consistent with microgliosis. The authors also noted transcripts indicative of reactive astrocytes in each brain region.

Another group (Belonwu et al.) performed two re-analyses of the data from Grubman et al. and Mathys et al. In one of their papers, they determined how cell-specific gene expression differed based on APOE genotype (Belonwu et al., 2022a). The authors compared individuals with the APOE3/3 and APOE3/4 genotype. The authors observed differential gene expression in individual cell types based on APOE genotype. For example, compared to APOE3/3 microglia, APOE3/4 microglia showed upregulated gliogenesis, myelination, cation transmembrane transport, and cellular projection pathways. The other study from Belonwu et al. re-analyzed the data from Mathys et al. and Grubman et al. by focusing on how sex affected cell-specific gene expression in AD (Belonwu et al., 2022b). The authors found differential cell-specific gene expression in AD depending on sex. For example, pathways involved in axonal sprouting were down-regulated specifically in microglia in AD male patients, while phosphodiesterase 4B pathways (which are involved in synaptic plasticity) were down-regulated specifically in microglia from female AD patients. These results demonstrate differential single-cell gene expression in AD based on APOE genotype and sex, and highlight the importance of assessing sex differences at the single cell level in neurobiological disease.

### 2.4. Autism

A few studies have examined single-cell gene expression in brains of autism spectrum disorder patients (Gandal et al., 2022; Velmeshev et al., 2019). A key study examined single-nucleus gene expression in the postmortem brains of people with ASD. (Velmeshev et al., 2019). They found genes involved in synaptic function, such as STX1A (downregulated), SYN2 (upregulated), and NRXN1 (upregulated), were differentially expressed in ASD L2/3 and L4 neurons compared to control neurons. Transcription factors involved in brain development, such as TCF25 (downregulated), SOX5 (upregulated), and RBFOX3 (downregulated) also had altered expression in L2/3 and L4 neurons from ASD samples. Further, microglia from ASD samples showed increased expression of microglial activation genes. Interestingly, the authors also found that certain genes in upper-layer projection neurons and microglia correlated with the clinical severity of ASD. Overall, these data identified differences in neuronal and glial gene expression in ASD brain compared to control brain. Another important study did not perform scRNA-seq on the brains of people with autism, but rather used previous scRNA-seq data to identify the cell subtypes expressing ASD risk genes (Wang et al., 2018). They found such genes were significantly enriched in inhibitory neurons, suggesting the pathology of ASD may involve dysfunction of inhibitory neurons.

### 2.5. Alcohol use disorders

One study performed snRNA-seq on the postmortem brains of patients suffering from alcohol dependence (Brenner et al., 2020). Differentially expressed genes (DEGs) were detected in every brain cell type with glial cells having the most DEGs. Some neuroinflammation genes were upregulated in astrocytes (e.g. - SLC1A3, FAS, MFGE8, IRF3). The authors also looked at non-coding RNAs in their study. The top DEGs in astrocytes was AC008957.2, a long non-coding RNA that is antisense to the SLC1A3 gene. SLC1A3 encodes a glutamate uptake transporter implicated in the neurobiology of substance use disorders. In alcohol dependent samples, both microglia and oligodendrocytes showed upregulated PTPRM, a gene encoding a tyrosine phosphatase. The authors also performed pathway analysis and found the GNRH signaling pathway to be the top overall upregulated pathway in alcohol dependence samples, specifically in astrocytes and microglia. Collectively, these studies suggest the existence of cell type specific transcriptional changes that can be linked to or may underlie alcohol use disorders.

### 2.6. Major depressive disorder

Nagy et al., 2020 performed snRNA-seq of the postmortem brains of people who died during an episode of major depressive disorder (MDD) (Nagy et al., 2020). Most DEGs were found in immature oligodendrocyte precursor cells (OPCs) and deep layer excitatory neurons. Some DEGs, including fatty acid desaturase 2 (FADS2), brain-type creatine kinase (CKB), and kazrin (KAZN), were previously identified in genome-wide association studies (GWAS) as being associated with MDD. CKB was downregulated in excitatory neurons in MDD and KAZN was upregulated in OPCs in MDD. Pathway analysis showed the DEGs in MDD tissue were involved in regulation of synaptic plasticity, fibroblast growth factor signaling, steroid hormone receptor chaperone cycling, the innate immune system, and cytoskeletal function. Taken together, these results highlight the cell types and pathways that may be involved in the pathophysiology of MDD.

## 3. Studies using human organoids

### 3.1. Intellectual disability/Autism

Multiple studies have used brain organoids to examine single-cell transcriptomes in models of ASDs (Table 2). One study examined the pathophysiology of a mutation in the autism susceptibility candidate 2 (AUTS2) gene using cerebral organoids (Fair et al., 2022). AUTS2 mutations can result in intellectual disability, microcephaly, seizures, and brain malformations. The authors collected peripheral blood mononuclear cells from a patient with a heterozygous AUTS2 mutation, reprogrammed these cells to induced pluripotent stem cells, and used them to generate cerebral organoids. scRNA-seq was performed on the resulting organoids. There was marked reduction in the proportion of a subtype of neural progenitor cells in AUTS2 organoids compared to control organoids. There was also altered cell cycle gene expression in AUTS2 organoids, consistent with the reduced growth seen in these organoids. Expression of genes involved in WNT pathway signaling, such as CTNNB1, were also reduced, consistent with altered proliferation of the AUTS2 cells. Paulsen et al. measured cell-specific gene expression in cortical organoid models of autism (Paulsen et al., 2022). The authors mutated three different autism-related genes (SUV420H1, ARID1B, and CHD8) in established human pluripotent stem cell lines and used them to generate cerebral cortical organoids. The SUV420H1 mutant organoids showed accelerated development of both GABAergic and deep-layer projection neurons. These mutant organoids also had upregulated expression of genes involved in neuronal maturation and synapse formation, such as KIF1B, DRAXIN, CAMK2N1. The ARID1B mutant organoids also contained increased numbers of GABAergic neurons during development in some cell lines, but decreased numbers of deep-layer projection neurons. Similar early maturation and increased numbers of GABAergic cells were seen in some of the CHD8 mutant organoids. Overall, these studies showed differential cell maturation and development of GABAergic cells in ASD organoids.

One study examined ASDs using forebrain organoids derived from patients carrying a homozygous CNTNAP2 mutation (de Jong et al., 2021). Homozygous loss-of-function mutations in CNTNAP2 cause a rare and severe neurodevelopmental syndrome that includes autism symptoms. Organoids with the CNTNAP2 mutation showed differential expression of autism-associated genes, such as CASK, ETFB, PBX1, and NR4A2, in excitatory neurons compared to excitatory neurons in control organoids. Another study used scRNA-seq to determine the effects of valproic acid on normal human forebrain organoids since valproic acid is associated with risk for ASDs (Meng et al., 2022). They found that valproic acid induced expression of ASD risk genes in excitatory neurons and immature neurons in the organoids. DEGs in excitatory neurons and immature neurons from the valproic acid-treated organoids were involved in functions such as neural development, synapse organization, and synaptic transmission. These studies collectively show differential expression of genes in control versus ASD model organoids.

### 3.2. Amyotrophic lateral sclerosis/frontotemporal dementia

One study examined organoid models of amyotrophic lateral sclerosis with frontotemporal dementia (ALS/FTD), a neurodegenerative disease characterized by cognitive decline and paralysis (Szebényi et al., 2021). They used induced pluripotent stem cells derived from patients with ALS/FTD that had a C9ORF72 hexanucleotide repeat expansion mutation that contributes to ALS/FTD. In their organoid models, they found that most DEGs were in astrocytes and deep-layer neurons. They identified DEGs such as C1QL1 in astrocytes and NRTK2 in neurons, which encode a complement subunit and the TrkB receptor, respectively. Both these genes are implicated in ALS pathogenesis. They also examined gene networks and found dysregulation of endoplasmic reticulum stress genes in astrocytes and DNA damage genes and cell death-related genes in neurons. Reactive astrocyte and inflammatory gene networks were also different in ALS/FTD organoids compared to control organoids. Overall, this study identified differently expressed genes and gene networks in astrocytes and neurons in organoid models of ALS/FTD.

### 3.3. Methamphetamine exposure

One study used cerebral organoids to study the effects of methamphetamine (METH) exposure on the transcriptomes of single-cells (Dang et al., 2021). Organoids were exposed to METH for one week. METH treatment downregulated genes involved in neural stem cell proliferation and neuronal differentiation. The genes most upregulated by METH were inflammation/immune, cytokine, and oxidative stress response genes, such as IL-6 and NLRP1. These genes were upregulated across most cell types, including astrocytes. Taken together, this study demonstrated an effect of METH on neuronal proliferation, survival, and immune/inflammation genes.

### 3.4. Psychosis

A few studies have used scRNA-seq of brain organoids to study psychotic disorders. One study used cerebral organoids to study the pathophysiology of schizophrenia (Scz) (Notaras et al., 2022). scRNA-seq of the Scz organoids showed decreased numbers of neurons and neural progenitor cells. There was also altered expression of POU5F1/OCT4, a transcription factor involved in cell differentiation, CRABP1, the proliferation and apoptosis balancing factor, and the inflammatory factor IFITM3. Scz progenitor cells down-regulated axon development pathways and enriched for structural, filament, adhesion, and angiogenic pathways. Furthermore, expression of the neurogenesis-related transcription factor POU3F2/BRN2 and the growth factor PTN were downregulated in both Scz progenitor cells and neurons. Collectively, these results suggest that dysregulated expression of cell growth and differentiation genes are seen in Scz progenitor cells and neurons.

Sawada et al. studied sc-RNA expression in cerebral organoid models of psychosis (Sawada et al., 2020). They collected peripheral blood mononuclear cells (PBMCs) from twin pairs in which one twin was diagnosed with a psychotic condition and the other was unaffected. These PBMCs were reprogrammed to induced pluripotent stem cells that were used to generate cerebral organoids. The cerebral organoids from the psychotic twin had significantly fewer proliferative progenitor cells and more GABAergic neurons that the organoids from the unaffected twin. Pathway enrichment analysis showed increased GABA receptor signaling genes and axon guidance genes in the affected twin. Furthermore, the transcription factors DLX1 and DLX2 that contribute to GABAergic neuronal specification were upregulated in organoids form the affected twin. There was also upregulation of genes associated with GABAergic synapses in the organoids from the affected twin. Taken together, these results suggest that differences in cell differentiation, specifically enhanced GABAergic specification during cortical development, may contribute to psychosis.

## 4. Discussion

In this review, we surveyed sc- and snRNA-seq studies performed on tissue from patients suffering from neurobiological disorders, including central nervous system neurological disorders and psychiatric diseases. This included investigation of postmortem human brains, as well as brain organoids derived from peripheral cells from patients with neurobiological disease. Identified reports covered a range of conditions, including epilepsy, cognitive disorders, mood disorders, and substance use disorders. They demonstrate that novel insights into neurobiological disease pathology can be garnered from scRNA-seq studies.

There are many ways in which scRNA-seq may improve our understanding or treatment of neurobiological disease, including discovery of novel cell types or subtypes involved in disease, identifying new pathophysiological mechanisms, or discovering novel drug targets or biomarkers. The studies reviewed here demonstrate examples of these. For example, Leng et al. found that a specific excitatory neuron subpopulation characterized by expression of RORB (RAR-related orphan receptor beta) was selectively vulnerable in AD. Also, Pfisterer et al. showed that principal neurons in layers L5–6 in particular showed marked gene dysregulation in epilepsy. Furthermore, Velmeshev et al., 2019 found L2/3 and L4 neurons specifically showed differential gene expression in ASD samples. Such insights into the specific cell types or subtypes involved in disease may improve our understanding or treatments of these conditions.

ScRNA-seq can also suggest new mechanisms of pathology. For example, Grubman et al. combined their snRNA-seq findings with data from GWAS studies to determine that 10 GWAS-identified genes for AD were dysregulated in AD astrocytes. These genes were all downstream of the TFEB transcription factor, which was also dysregulated in AD astrocytes. These results identify novel dysregulated pathways in astrocytes in AD. Also, Belonwu et al., 2022a found that microglia and oligodendrocytes showed opposite patterns of differential gene expression between APOE3/3 AD cells and APOE3/4 AD cells, suggesting different mechanisms of neurodegeneration based on genotype. Similarly, Belonwu et al., 2022b found different patterns of glial gene expression in men vs women in AD, suggesting different mechanisms of neurodegeneration based on sex. Such information may inform personalized medicine treatment strategies.

ScRNA-seq may also suggest novel treatment targets or new biomarkers. For example, in the study by Pfisterer et al., the majority of dysregulated genes identified had not been previously reported for epilepsy. Such genes may represent novel treatment targets. Also, Kumar et al. propose that their data suggest immunomodulatory treatments could be used for epilepsy. For example, they noted that genes involved in adhesion and trafficking of leukocytes (such as integrins) were increased in epileptic tissue, suggesting infiltration of peripheral immune cells may contribute to disease pathogenesis. They speculate that treatment with compounds that inhibit leukocyte migration may help treat epilepsy. The study by Velmeshev et al. is particularly insightful with regard to novel treatment targets, as it correlates cell-specific gene expression with clinical symptoms from the patients during their lifetime. In doing so, genes were identified (e.g. – FZD3 and CHL1 in L2/3 neurons and NCK2 in microglia) whose protein products could potentially be targeted in future studies to see if doing so improves symptoms in patients. Correlating cell-specific gene expression with clinical symptoms should be performed in other single-cell studies to generate specific targets for future studies. Regarding potential identification of biomarkers, Jakel et al. found that different MS lesions subtypes could be identified by different gene expression. Overall, these studies provide examples of how scRNA-seq of brain tissue from patients with neurobiological conditions may provide new treatment targets or biomarkers.

Additional broader insights into neurobiological disease may be gained from these studies. For example, imbalance between excitatory and inhibitory neurons is a common theme of these studies. While this may be expected in the context of a disease such as epilepsy, it is also seen in Alzheimer’s, psychosis, and autism. Leng et al. found decreased numbers of excitatory neurons in Alzheimer’s disease (Leng et al., 2021), while Sawada et al. and Paulsen et al. found increased numbers of GABAergic neurons in schizophrenia and autism, respectively (Paulsen et al., 2022; Sawada et al., 2020). A fine-tuned balance between excitation and inhibition is essential for proper brain function, which may explain why dysregulation of this process underlies such varied diseases. Another theme is the importance of glial cells in neurobiological conditions. Most current medications for neurobiological disease target neuronal function by modulating neurotransmitter levels or acting on neurotransmitter receptors; however, the results of these studies suggest targeting glial cells may be a novel way to improve treatment. For example, activated/inflammatory phenotypes of microglia and/or astrocytes are observed in epilepsy, Alzheimer’s, autism, and substance use disorders. Gene dysregulation of oligodendrocytes/myelination was also noted in Alzheimer’s and MDD. A final theme is that many conditions involved changes in the neuroimmune system. Indeed, most conditions examined, including epilepsy, Alzheimer’s, autism, and substance use disorders, were associated with changes in immune genes, such as increased expression of inflammatory cytokines or markers of microglial activation. Such insights may inform novel treatment strategies for neurobiological disease.

Each of the two main approaches (postmortem brains vs organoids) used in these reviewed studies has advantages and disadvantages. Unlike the organoids, the postmortem brains are real human brains rather than models; however, the data from human brains is likely more variable. Each brain, when alive, was exposed to different environmental conditions that would likely have different effects on single-cell transcriptomes. Also, the postmortem diseased brains had likely been exposed to various psychotropic medications whereas the control brains likely were not. Exposure to psychotropic medications likely impacts single-cell gene expression. Unlike postmortem brains, organoids allow for investigation of developmental components of diseases, such as autism and schizophrenia. There is also less variability with organoids and they can undergo targeted manipulations, such as genetic engineering to determine the effects of specific mutations on single-cell gene expression. However, organoids do not model the full complexity of a real human brain. The organoids lack multiple, interconnected brain regions, a peripheral nervous system, and sensory input from an external environment. As such, there are significant weaknesses of using brain organoids for these types of studies. The strengths of both approaches can be used to complement each other to understand further the neurobiology of these diseases.

These studies also demonstrate how scRNA-seq has advantages over previous methods for understanding these diseases. Techniques such as bulk RNA seq detect gene expression averaged across millions of cells. Therefore, genes involved in disease processes may be highly upregulated in a certain cell type, but this increased expression might not be detected as significant when averaged with other cells in the tissue in which the gene is not upregulated. Brenner et al. provides an illustration of this when they compared their postmortem alcoholic brain snRNA-seq results to bulk RNA seq results. They found that among the 33 genes differentially expressed in microglia in their study, only the gene with the highest fold change (SLC11A1) was detected as significant in bulk RNA seq analyses. This demonstrates how scRNA-seq can identify new transcripts potentially involved in disease. Also, many of the studies reviewed here performed snRNA-seq instead of scRNA-seq. Each method has different advantages and disadvantages. ScRNA-seq captures transcripts present in the cytoplasm as well as the nucleus; however, it is best done with fresh tissue. By contrast, snRNA-seq, while it measures fewer transcripts, can be done on frozen tissue, which is why it was the method of choice for the studies of postmortem human brains. Taken together, scRNA-seq studies of postmortem brains and organoid models could provide significant insights into disease processes and potential treatments.

It is worth assessing and comparing the relative quality of these studies. The quality of sc- or snRNA-seq studies can be evaluated using various metrics such as number of samples, number of cells sequenced, and methods of quality control. The studies by Mathys et al. and Belonwu et al. had high sample numbers - 24 control brains and 24 diseased brains for Mathys et al. and 30/27 control brains and 30/26 diseased brains for the two studies by Belonwu et al. The studies with the least number of samples include Brenner et al. with 4 control brains and 3 disease brains and Jakel et al. with 4 control brains and 5 diseased brains. Velmeshev et al. and Pfisterer et al. had the highest number of cells sequenced, with 104,559 cells and 101,982 cells sequenced, respectively. Alternatively, Grubman et al. and Brenner et al. sequenced the least number of cells with 13,214 and 16,305 cells, respectively. Studies also varied in their quality control. Quality control during sc- or snRNA-seq data analysis is important for generating high quality data and usually involves the following steps (Hong et al., 2022). Cells with unusually low or high numbers of transcripts are often removed from analysis because they likely represent ambient RNA or multiple cells (i.e. – doublets), respectively. Also, cells are usually filtered based on the percentage of their transcripts that are mitochondrial. Cells with a high percentage of mitochondrial transcripts are thought to represent stressed or dying cells and are therefore removed. Most studies were similar in their level of quality control in that they excluded cells with both very low or very high numbers of transcripts, as well as cells with a high percentage of mitochondrial transcripts. Brenner et al. excluded cells with >20% mitochondrial transcripts, but it was unclear whether they excluded cells with unusually low or high numbers of transcripts. Importantly, all of the studies discussed here have made their data publicly available (Table 1). Some data are protected under different forms of access control, but are available upon request. Furthermore, some of these studies were performed entirely on publicly available datasets, demonstrating the importance of data accessibility, as well as the importance of the availability of comprehensive study metadata and analytical pipelines for single-cell resolution datasets. Overall, future studies would benefit from examining a large number of samples, sequencing many cells, using the most up-to-date and rigorous quality control practices, and making data publicly available. (See Table 1.)

### 4.1. Future directions

**Fig. 1.**
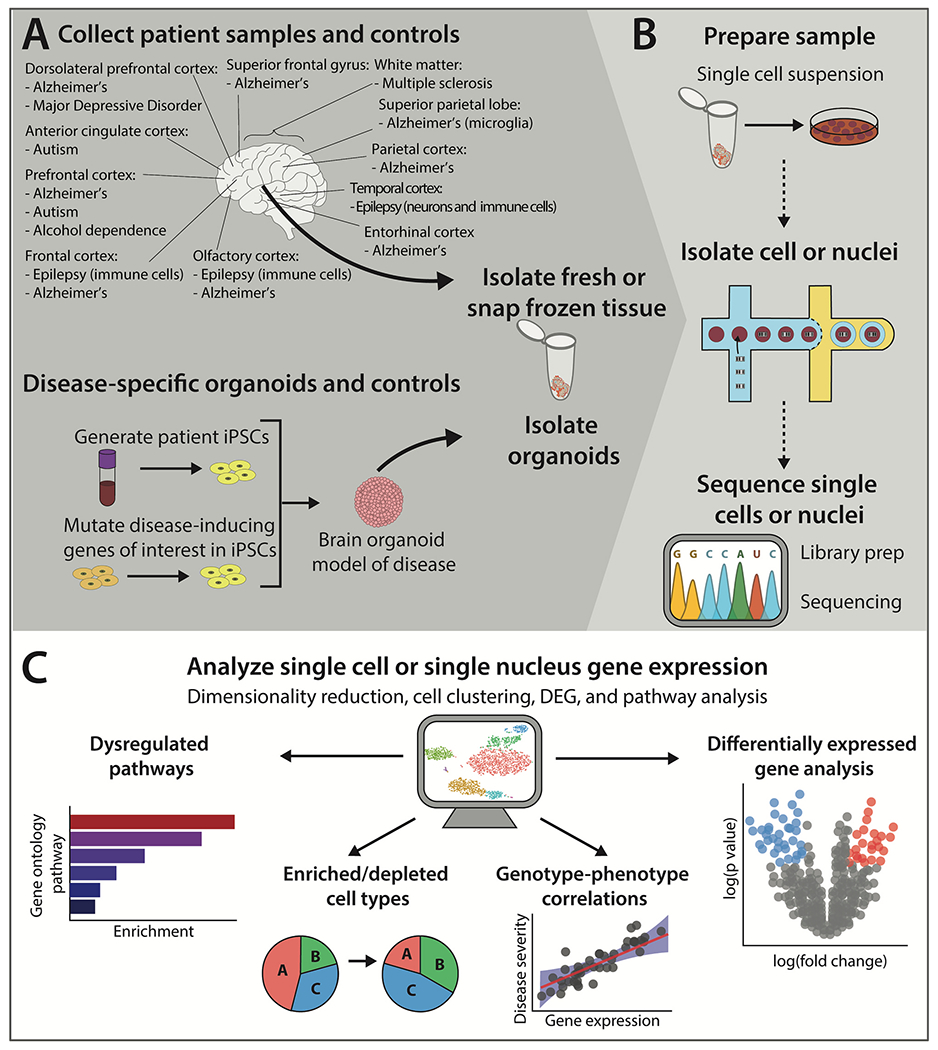
A diagram of sc- or snRNA-seq studies in the tissues of people with neurobiological disorders. A.) Brain regions in which sc- or snRNA-seq have been performed are outlined and the neurobiological disorders that have been studied in those brain regions are listed below each region. Many scRNA-seq studies were also performed on organoids derived from induced pluripotent stem cells (iPSCs) from patients. B.) Cells from either post-mortem brains or organoids are processed for scRNA-seq and the transcriptomes are sequenced. C.) Analysis of scRNA-seq data provides information on dysregulated gene pathways, enriched or depleted cell types, genotype-phenotype correlations, and/or differentially expressed genes in tissue from people suffering from neurobiological disorders.

Many neurobiological diseases involve dysfunction in multiple brain regions. Most of the studies reviewed here only examined tissue from one or a few brain regions, and the majority of examined samples were cortical (Fig. 1). However, other brain regions are involved in the pathogenesis of neurobiological conditions. Notably, the amygdala, striatum, and hippocampus are not well-represented in current scRNA-seq studies of neurobiological disease of the human brain. Examining these other brain regions is necessary for a more complete understanding of these conditions at the single-cell level. Also, while some conditions, such as AD, are well-represented in the reviewed studies, other conditions have received relatively little attention. For example, while our search efforts were not exhaustive, we did not identify any reports examining attention deficit hyperactivity disorder, bipolar disorder, anxiety disorders, obsessive-compulsive disorders, eating disorders, or sleeping disorders. Also, some conditions had only one or a few studies, such as major depressive disorder, alcohol use disorders, and MS.

Other future directions could include combining scRNA-seq with single-cell proteomics. Some studies find that the correlation between transcript levels and protein level is not always significant (Lemée et al., 2018; Payne, 2015), and therefore single-cell proteomics may be a better representation of cell function/phenotype. Such information could be especially useful for drug discovery because it is usually protein, not mRNA, that is the target of most therapies. Data from the reviewed studies could also be useful comparisons for other studies. For example, studies in brain cancer find that neuronal activity influences tumor progression - for review, see (Pan and Monje, 2022). The control data in these studies could be a useful comparison for studies of the transcriptomes of neurons interacting with tumors. Furthermore, some of the studies only used a few samples per condition or analyzed a relatively small number of cells. Future studies should improve study quality by including more samples and analyzing more cells. Also, the studies used different quality control methods, so re-analysis of some datasets using the most rigorous practices would be beneficial. Future work should also use the most rigorous, up-to-date analytic methods consistently across studies. It would advance the field to make all data from postmortem sc- or snRNA-seq studies publicly available. Doing so could allow for collaborations between groups and meta-analyses of the data from diseased tissue. Finally, many of these studies highlighted patterns in the data that provided interesting descriptions of disease pathology, but they did not make strong and clear recommendations for specific proteins or pathways to follow up on as potential novel treatment targets. Future sc- or snRNA-seq studies could determine which specific dysregulated transcripts are also druggable targets. Overall, there are many open areas for further research in this field.

## 5. Summary

In this review, we surveyed sc- and snRNA-seq studies performed on tissue from patients suffering from neurobiological conditions. This included postmortem human brains, as well as brain organoids derived from peripheral cells from patients. Identified reports covered a range of conditions, including epilepsy, cognitive disorders, mood disorders, and substance use disorders. We highlighted how these previous sc- or snRNA-seq studies have provided novel insights into the pathophysiology of these conditions. We argue that additional sc- or snRNA-seq studies of neurobiological disease could advance our treatment of these conditions by identifying potential therapeutic targets that are not easily revealed when measuring transcriptional changes at the bulk level.

## Figures and Tables

**Table 1:** Single-cell or Single-nucleus RNA sequencing studies on human post-mortem brain tissue in people with neurobiological disorders.

Study	Condition Studied	N	Brain Regions; Cell Types; Number of Nuclei/Cells Analyzed	Platform	Main Findings
Pfisterer et al. (2020)	Epilepsy	10 controls, 9 temporal lobe epilepsy samples	Temporal cortex; Neurons; 101,982 cells	10× Genomics; Smart-seq2	Genes involved in hyperexcitability, such as glutamate receptors, neurotransmitter synthesis genes, etc. were altered in epilepsy neurons
Kumar et al. (2022)	Epilepsy	Number of control samples not specified, 6 epilepsy samples	Olfactory, frontal, and temporal lobes; Immune cells; 85,780 cells	10× Genomics	Increased pro-inflammatory gene expression in microglia; Infiltration of activated, cytotoxic leukocytes
Jäkel et al. (2019)	Multiple sclerosis	5 controls, 4 MS samples	White matter (brain region not specified); All cell types; 17,799 cells	10× Genomics	Altered oligodendrocyte subclusters and upregulation of myelin-related genes in MS oligodendrocytes
Grubman et al. (2019)	Alzheimer’s	6 controls, 6 AD samples	Entorhinal cortex; All cell types; 13,214 cells	10× Genomics	Upregulation of stress response genes in all cell types; downregulated synapse-associated genes in neurons; increased immune gene expression in microglia, astrocytes, and endothelial cells; identification of cell-specific AD gene regulatory networks
Mathys et al., 2019	Alzheimer’s	24 controls, 24 AD samples	Prefrontal cortex (Brodmann area 10); All cell types; 80,660 cells	10× Genomics	Altered myelination-related gene expression in multiple cell types; Upregulation of microglial and astrocyte activation genes; Increased global stress response gene expression in all cell types in late-stage AD
Zhou et al. (2020)	Alzheimer’s	11 controls, 11 AD with TREM2-common variant, 10 AD with TREM2-R62H; 5 AD with TREM2-R47H and 5 AD with TREM2-common variant	Dorsolateral prefrontal cortex/parietal cortex; All cell types; 73,419 cells	10× Genomics	Decreased expression of microglial activation genes in TREM2-R62H and R47H microglia compared to TREM2 common variant microglia in AD
Leng et al. (2021)	Alzheimer’s	10 samples: 3 Braak stage 0, 4 Braak stage 2, 3 Braak stage 6	Entorhinal cortex/ superior frontal gyrus; All cell types; 42,528 cells from EC and 63,608 cells from SFG	10× Genomics	Reduced numbers of excitatory neurons in late-stage AD; increased microglial numbers
Belonwu et al. (2022a)	Alzheimer’s	30 controls, 30 AD samples	Prefrontal cortex/ entorhinal cortex; All cell types; 70,634 cells from PFC and 13,214 cells from EC	10× Genomics	Differential cell-specific gene expression in AD based on APOE genotype
Belonwu et al. (2022b)	Alzheimer’s	27 controls, 26 AD samples	Prefrontal cortex/ entorhinal cortex; All cell types; 70,634 cells from PFC and 13,214 cells from EC	10× Genomics	Differential cell-specific gene expression in AD based on sex
Velmeshev et al. (2019)	Autism	16 controls, 15 ASD samples	Prefrontal cortex, anterior cingulate cortex; All cell types; 104,559 cells	10× Genomics	Differential expression of genes involved in synaptic function and brain development in ASD neurons; differential expression of activation genes in ASD microglia
Brenner et al. (2020)	Alcohol dependence	4 controls, 3 alcohol dependence samples	Prefrontal cortex; All cell types; 16,305 cells	10× Genomics	Glial cells showed the most differentially expressed genes, including neuroinflammation genes
Nagy et al. (2020)	Major Depressive Disorder	17 controls, 17 MDD samples	Dorsolateral prefrontal cortex; all cell types; 78,886 cells	10× Genomics	Oligodendrocyte precursor cells and deep excitatory neurons showed the most differentially expressed genes; genes involved in synaptic plasticity, cell signaling, the innate immune system, and cytoskeletal function were most dysregulated

Study	Quality Control	Data Availability
Pfisterer et al. (2020)	• Cells with >8% mitochondrial genes removed • Doublet cells removed	European Genome-phenome Archive (EGA): EGAS00001002882.
Kumar et al. (2022)	• Cells with <300 genes or > 5000 genes removed • Cells with >20% mitochondrial genes removed	Gene Expression Omnibus (GEO): GSE201048 Additional: Raw counts and analyzed R objects are also available at https://epicimmuneatlas.org/NatNeu2022. Associated data used to produce figures are also deposited in the Zenodo repository (https://doi.org/10.5281/zenodo.6477100).
Jäkel et al. (2019)	• Cells with <200 genes removed • Cells with >20% mitochondrial genes removed	European Genome-phenome Archive (EGA): EGAS00001003412. Gene Expression Omnibus (GEO): GSE118257. Additional: A browsable web resource is available at https://ki.se/en/mbb/oligointernode.
Grubman et al. (2019)	• Cells outside 5th and 95th percentile with respect to number of total and unique genes were removed • Cells with >10% mitochondrial genes removed	Gene Expression Omnibus (GEO): GSE138852. Additional: Data can also be visualized via the interactive web application at adsn.ddnetbio.com. Single-cell gene expression data and metadata can also be downloaded directly via adsn.ddnetbio.com.
Mathys et al., 2019	• Cells with <200 genes removed • Cells with abnormally high ratio of mitochondrial genes removed	The Rush Alzheimer’s Disease Center (RADC) Research Resource Sharing Hub: https://www.radc.rush.edu/docs/omics.htm (snRNA-seq PFC) Synapse: (https://www.synapse.org/#!Synapse:syn18485175) under the doi https://doi.org/10.7303/syn18485175. The Religious Orders Study and Memory and Aging Project (ROSMAP) metadata can be accessed at https://www.synapse.org/#!Synapse:syn3157322. The data are available under controlled use conditions set by human privacy regulations. To access the data, a data use agreement is needed.
Zhou et al. (2020)	• Cells with >5% mitochondrial genes removed • Cells with <400 genes or > 7000 genes removed	Murine data: Gene Expression Omnibus (GEO): GSE140511 Human data: AD Knowledge Portal (https://adknowledgeportal.org) under study snRNAseqAD_TREM2 and are also accessible through Synapse at https://doi.org/10.7303/syn21125841. Additional ROSMAP data can be requested at https://www.radc.rush.edu/.
Leng et al. (2021)	• Cells with <200 unique genes excluded	Gene Expression Omnibus (GEO): GSE147528. Additional: Processed data is available at Synapse.org under the Synapse ID syn21788402 and can also be explored interactively through the CellXGene platform at https://kampmannlab.ucsf.edu/ad-brain. Data from Mathys et al. (above) was also used in this study.
Belonwu et al. (2022a)	• Cells outside 5th and 95th percentile with respect to number of total and unique genes were removed • Cells with >10% mitochondrial genes removed	The authors utilized previously publicly available datasets in this study. Prefrontal cortex from the Accelerating Medicines Partnership Alzheimer’s Disease Project (AMP-AD) Knowledge Portal under the Religious Orders Study and Memory and Aging Project (ROSMAP) (https://www.synapse.org/#!Synapse:syn18485175 and https://www.synapse.org/#!Synapse:syn3157322). Entorhinal cortex from a data repository provided by Grubman et al. (2019) (http://adsn.ddnetbio.com/). Data from the entorhinal cortex may also be accessed from the Gene Expression Omnibus under the accession number GSE138852. Access to the prefrontal cortex dataset requires a formal request to ROSMAP.
Belonwu et al. (2022b)	• Cells outside 5th and 95th percentile with respect to number of total and unique genes were removed • Cells with >10% mitochondrial genes removed	See above.
Velmeshev et al. (2019)	• Cells with unique number of genes <500 removed • Cells with >5% mitochondrial genes removed	Sequence Read Archive (SRA): PRJNA434002. Additional: Analyzed data and visualization are available at https://autism.cells.ucsc.edu.
Brenner et al. (2020)	• >20% mitochondrial genes removed	Gene Expression Omnibus (GEO): GSE141552.
Nagy et al. (2020)	• Cells with fewer than 110 genes removed • Cells with top 0.5% number of genes removed • Other custom filtering	Gene Expression Omnibus (GEO): GSE144136.

**Table 2:** Single-Cell RNA sequencing studies on brain organoids derived from patients with neurobiological disorders.

Study	Methods	Condition Modeled	Main Findings
Fair et al. (2022)	Peripheral blood mononuclear cells from patient were reprogrammed to induced pluripotent stem cells which were used to generate cerebral organoids	Heterozygous AUTS2 mutation	Decreased proportion of neural progenitor cells in AUTS2 organoids; Altered cell cycle and WNT pathway signaling genes in AUTS2 organoids
Paulsen et al. (2022)	Established induced pluripotent stem cell lines were genetically modified using CRISPR to mutate genes of interest; engineered cell lines were then grown into cortical organoids	Autism (via SUV420H1, ARID1B, and CHD8 mutations)	Accelerated maturation and increased numbers of GABAergic neurons in autism model organoids
Szebényi et al. (2021)	Induced pluripotent stem cell lines from patients with ALS/FTD were used to generate organoids	ALS/FTD	Dysregulated activation, immune, and stress genes in astrocytes and DNA damage genes and cell death-related genes in neurons from ALS/FTD organoids
Dang et al. (2021)	Human embryonic stem cells were used to generate cerebral organoids which were then exposed to methamphetamine	Methamphetamine exposure	Down-regulated proliferation and differentiation genes in neurons; up-regulated inflammation/immune, cytokine, and oxidative stress response genes in METH organoids
Notaras et al. (2022)	Induced pluripotent stem cell lines from patients with schizophrenia were used to generate cerebral organoids	Schizophrenia	Dysregulated expression of transcription factors involved in cell differentiation and growth factor pathway genes in Scz organoids
Sawada et al. (2020)	Peripheral blood mononuclear cells were isolated from monozygotic twins, one of which had a psychotic illness and the other of which was unaffected. Induced pluripotent stem cells were generated from PBMCs and these were used to generate cerebral organoids	Psychosis	Psychosis organoids had fewer proliferative progenitor cells and more GABAergic neurons, as well as increased expression of GABA signaling and GABA synapse genes

## Data Availability

No data was used for the research described in the article.
